# Genome-wide association study for resistance in bread wheat (*Triticum aestivum* L.) to stripe rust (*Puccinia striiformis* f. sp. *tritici*) races in Argentina

**DOI:** 10.1186/s12870-022-03916-y

**Published:** 2022-11-24

**Authors:** M. F. Franco, A. N. Polacco, P. E. Campos, A. C. Pontaroli, L. S. Vanzetti

**Affiliations:** 1 Facultad de Ciencias Agrarias, Universidad Nacional de Mar del Plata, 7620 Balcarce, CP Argentina; 2 Consejo Nacional de Investigaciones Científicas Y Técnicas (CONICET), Buenos Aires, Argentina; 3 Estación Experimental Agropecuaria INTA Balcarce, 7620 Balcarce, CP Argentina; 4 Estación Experimental Agropecuaria INTA Bordenave, 8187 Bordenave, CP Argentina; 5 Estación Experimental Agropecuaria INTA Marcos Juárez, 2580 Marcos Juárez, CP Argentina

**Keywords:** Genome-wide association study (GWAS), Single nucleotide polymorphism (SNP), Adult plant resistance (APR), All-stage resistance (ASR)

## Abstract

**Background:**

Wheat stripe rust, caused by *Puccinia striiformis* f. sp. *tritici* (*Pst*), is one of the most devastating diseases of the wheat crop. It causes significant reductions in both grain yield and grain quality. In recent years, new and more virulent races have overcome many of the known resistance genes in Argentinian germplasm. In order to identify *loci* conferring resistance to the local races of *Pst* for effective utilization in future breeding programs, a genome-wide association study (GWAS) was performed using a collection of 245 bread wheat lines genotyped with 90 K SNPs.

**Results:**

To search for adult plant resistance (APR) the panel was evaluated for disease severity (DS) and area under disease progress curve (AUDPC) in field trials during two years under natural infection conditions. To look for seedling or all-stage resistance (ASR) the panel was evaluated to determine infection type (IT) under greenhouse conditions against two prevalent races in Argentina. The phenotypic data showed that the panel possessed enough genetic variability for searching for sources of resistance to *Pst*. Significant correlations between years were observed for *Pst* response in the field and high heritability values were found for DS (H^2^ = 0.89) and AUDPC (H^2^ = 0.93). Based on GWAS, eight markers associated with *Pst* resistance (FDR < 0.01) were identified, of these, five were associated with ASR (on chromosomes 1B, 2A, 3A and 5B) and three with APR (on chromosomes 3B and 7A). These markers explained between 2% and 32.62% of the phenotypic variation. Five of the markers corresponded with previously reported *Yr* genes/QTL, while the other three (*QYr.Bce.1B.sd.1*, *QYr.Bce.3A.sd* and *QYr.Bce.3B.APR.2*) might be novel resistance *loci*.

**Conclusion:**

Our results revealed high genetic variation for resistance to Argentinian stripe rust races in the germplasm used here. It constitutes a very promising step towards the improvement of *Pst* resistance of bread wheat in Argentina. Also, the identification of new resistance *loci* would represent a substantial advance for diversifying the current set of resistance genes and to advance in the improvement of the durable resistance to the disease.

**Supplementary Information:**

The online version contains supplementary material available at 10.1186/s12870-022-03916-y.

## Background

Bread wheat (*Triticum aestivum* L.) is an important crop for global food security that provides 20% of the daily calories and over 25% of the protein consumed by the human population [1]. In spite of the fact that more than 700 million tons of wheat are produced every year in the world, further production increases are required to satisfy the future demand for food [2]. Argentina is among the countries with the capacity to fulfill part of that demand [1]. This country has the potential to produce five times the grains required by its population [3], because of the Rolling Pampas, one of the most productive regions of the world [4]. However, wheat production faces several biotic and abiotic obstacles that usually cause major economic losses in terms of yield and quality. Currently, estimates of potential yield losses for the wheat crop attributed to pathogens are estimated to be around 21.5% [5]. Among the main diseases described in wheat, rusts take a preponderant place, as they are widespread in all wheat regions and are highly destructive [6].

Stripe (yellow) rust, caused by the biotrophic fungus *Puccinia striiformis* Westend. f. sp. *tritici* Erikss (*Pst*) is one of the most devastating diseases of the wheat crop worldwide [7–9]. Stripe rust causes significant reductions in both yield and grain quality. Yield losses can range from 10 to 70% depending on the susceptibility of the cultivar, timing of initial infection, rate of disease development, and duration of disease [10]. However, *Pst* can cause 100% of yield losses in highly susceptible genotypes if infection occurs very early and the disease continues developing during the entire growing season [10, 11].

Historically, *Pst* epidemics have occurred mainly in temperate areas with cool and wet weather conditions [12]. Until a few years ago, stripe rust was considered a sporadic disease in Argentina. In fact, it used to occur in the Southern wheat region only when temperatures during spring were lower than normal and under high humidity conditions. However, since 2015 both the incidence and severity of this disease have been increasing [13], and in 2017, the disease reached an epiphytic level in the Argentine wheat region [14, 15]. The basis of this abrupt change seems to be the wide spread of susceptible cultivars and the emergence of new races that have expanded their virulence profiles, capable of adapting to warmer temperatures, and with higher aggressiveness than the previously characterized races [15]. These races overcame many of the major resistance genes in germplasm adapted to Argentina [16, 17].

Although some fungicides are effective for the control of stripe rust, the chemical application adds considerable cost to crop production and poses potential environmental risks. For this reason, the use of genetic resistance represents the most effective, economic, and ecological strategy to reduce losses due to this disease [18]. Genetic resistance to stripe rust can be broadly categorized as either race-specific or non-race-specific [18, 19]. The former is often referred to as seedling resistance or all-stage resistance (ASR) because it can be detected at the seedling stage but remains effective at all stages of plant growth. This kind of resistance generally results in a strong hypersensitive response associated with high levels of resistance. It is frequently conferred by single genes or combinations of a few genes and is characterized by being specific to the race of the pathogen [19]. In contrast, non-race-specific resistance, also called adult plant resistance (APR), is expressed at later stages of plant growth through low disease severities at the adult-plant stage in the field [19]. This type of resistance is polygenic and governed by the additive effects of several low-effect genes [20]. These genes are effective against all *Pst* races and are characterized by various degrees of resistance (partial or quantitative resistance) [18, 21–23].

Due to the high level of resistance and the easy incorporation of single genes into commercial cultivars, ASR has been more attractive to breeding programs in the past [24]. However, this type of resistance is readily broken by new virulent races; hence, it has a relatively limited effective life [25–27]. Contrarily, APR has the advantage of providing more durable resistance [21, 28]. However, cultivars with this type of resistance can suffer significant yield losses when rust starts developing early in the growing season and environmental conditions continue to be favorable for the expression of the disease [7]. This situation has forced wheat breeders to focus on pyramiding strategies that combine multiple race-specific and non-race-specific resistance genes. This approach provides a complex resistance against the dynamics of pathogen virulence and increases the durability of the deployed resistance [29, 30].

According to the Catalogue of Gene Symbols for Wheat [31] and the 2020 Supplement (https://wheat.pw.usda.gov/GG3/wgc) there are currently 83 officially designated *Yr* genes for resistance to stripe rust and 42 temporarily designated genes. Many of these genes have been introduced into wheat varieties from wheat relatives and exotic species. However, most of them are already ineffective against the new *Pst* races that appeared in the last years [14, 15]. Besides, numerous quantitative trait loci (QTL) studies have been carried out identifying > 350 QTL so far [32]. Nevertheless, most of the previous research studies, were based on classical mapping methods that are costly, characterized by low resolution in QTL detection, and restrict the number of alleles sampled per locus in each population hindering the examination of the full range of genetic diversity available in the crop [33, 34]. In this way, considering that *Pst* constitutes a great threat in the Argentine wheat region, the identification and characterization of new sources of resistance to the local races have become essential to improve durable resistance to this disease.

Currently, the possibility of obtaining resistance gene combinations is being increasingly facilitated by the recent advances in genomics, statistics, and efficient mechanisms of genome manipulation [35]. Using efficient methods of genetic analysis to facilitate the identification and pyramiding of genomic regions associated with traits of agronomic importance in diverse germplasm accessions is an aim for the effective use of diversity in crop breeding programs. Genome-wide association study (GWAS) constitutes a widely used approach to detect quantitative trait *loci* (QTL) in plants, as they combine phenotypic and genotypic data from a large number of individuals [36]. This tool can examine a relatively wide portion of natural variation in a species and detect trait associations to much smaller genomic regions because the sampled diversity includes many more recombination events than those observed in traditional recombinant inbred line or doubled haploid populations [37]. GWAS has been used in QTL mapping for diverse traits in numerous plant species [38, 39]. In wheat, GWAS has been successfully used for agronomic traits [40, 41], quality [42], and disease resistance [43, 44], among others. Therefore, GWAS represents a highly advantageous alternative for the identification and characterization of new sources of durable resistance to *Pst*.

In order to advance in the genetic improvement of the resistance to stripe rust, the objectives of this study were to: (1) characterize the diversity of the resistance to local races of stripe rust of Argentina in a wide collection of spring bread wheat genotypes, (2) to conduct a genome-wide search for single nucleotide polymorphisms (SNPs) associated with resistance to current races of *Pst* from Argentina and (3) to compare the *Pst* resistance *loci* identified in this work with previously identified *Yr* genes and QTs.

## Results

### Evaluation of wheat germplasm

**Fig. 1:**
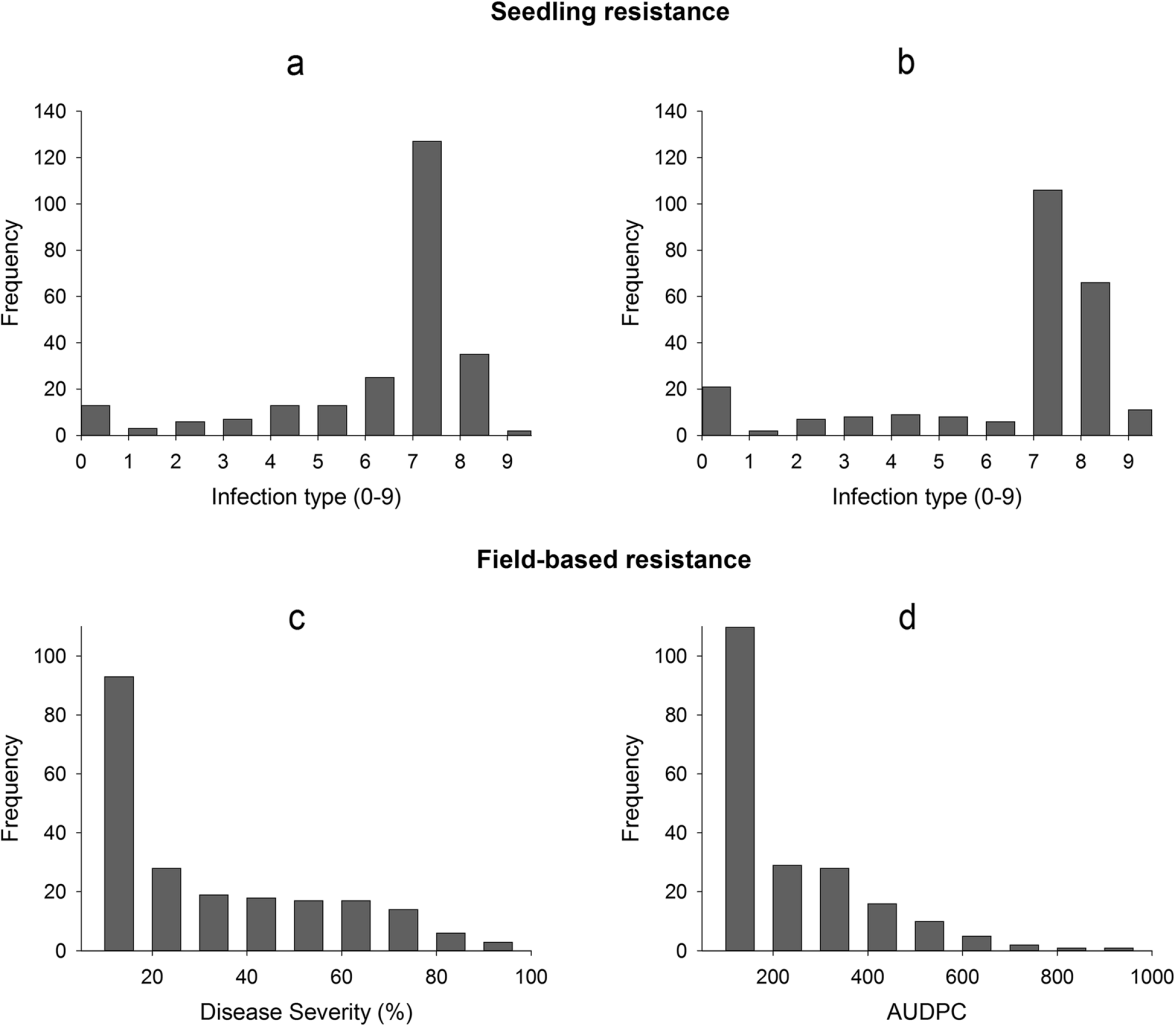
Frequency distribution of the response to stripe rust in the collection of 245 spring bread wheat genotypes evaluated for seedling resistance -infection type (IT) for the races **a** *Yr19-71* and **b** *Yr20-161-* and field-based resistance -**c** disease severity (DS) and **d** area under disease progress curve (AUDPC)-

Wheat germplasm responses (ITs) to two *Pst* races of stripe rust pathogen at seedling stage and the DS and AUDPC data of the adult-plant stage evaluated in the field during the two years are summarized in Table 1. The mapping population showed diverse responses to stripe rust and continuous variation was observed for all the variables across the conducted experiments (Fig. 1). At the seedling stage, broad phenotypic variation was exhibited among the 245 genotypes evaluated for each race (*P* < 2.16 × 10^–16^) (Table S1). Based on the IT data, for *Yr19-71*, 17.2% of the accessions displayed high resistance reactions (IT = 0–4), while 67.2% of the accessions were considered susceptible (IT = 7–9). The remaining 15.6% of the accessions showed an intermediate reaction (IT = 5–6). On the other hand, for *Yr20-161*, 19.3% of the accessions exhibited a resistant response, 75% of the accessions were highly susceptible and 5.7% of the genotypes displayed an intermediate reaction (Fig. 1).

**Table 1:** Means, minimum, maximum and standard deviations for *Pst* resistance in the collection of bread wheat

Resistance type	Trait	Trial	Mean	Minimum	Maximum	standard deviations (S.D.)
Seedling resistance	IT	*Yr19-71*	6.10	0.00	9.00	2.10
		*Yr20-161*	6.15	0.00	9.00	2.55
Field-based resistance	DS	2020	34	5	100	30
		2021	20	1	88	19
		Both years	27	1	100	26
	AUDPC	2020	172.08	2	960.25	177.45
		2021	140.39	2	720.25	160.44
		Both years	156.31	2	926.25	169.73

In the APR screening, sequential restricted maximum likelihood ratio tests revealed highly significant variation due to genotypes (*P* < 0.0001) (Table S2). Across the years, DS ranged from 0.05 to 1 across the panel, with an average of 0.27. The AUDPC ranged from 2 to 926.25 with an average of 156.31. Variance component analysis by restricted maximum likelihood (REML) showed that σ^2^_g_ was greater than σ^2^_ge_ for both variables. High broad-sense heritability values were observed for DS (H^2^ = 0.89) and AUDPC (H^2^ = 0.93) indicating that a high portion of the observed phenotypic variation was caused by the genotypic component (Table S3).

**Fig. 2:**
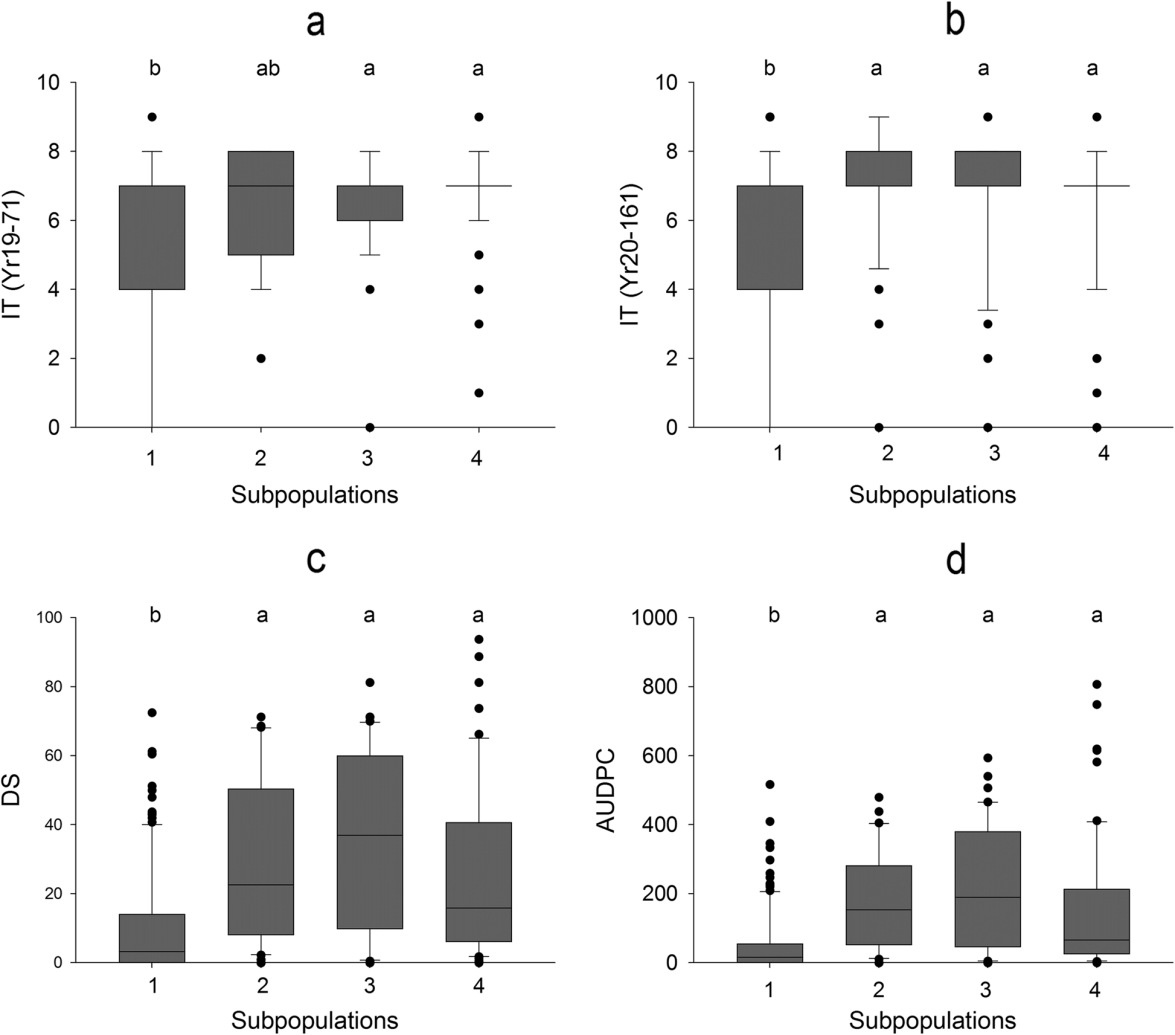
Effect of population structure on the response to stripe rust in the collection of 245 spring bread wheat genotypes: **a** Infection type (IT) for the race *Yr19-71*, **b** IT for the race *Yr20-161*, **c** Disease Severity (DS), and **d** Area Under Disease Progress Curve (AUDPC). Box plots show trait distribution and compare the levels of stripe rust among the four subpopulations. Boxes indicate the middle 50% of the data and the median (solid horizontal line). The whiskers show the range of adjacent values; dots indicate outliers. The subpopulation represented by the same lowercase letter(s) are not significantly different at *p* = 0.01

Based on the population structure analysis performed in this population by Zhang et al. [45] (Fig S1), a significant correlation (*P* < 0.001 and r ranging from 0.19 to 0.34) was observed between the population sub-clusters and the response to stripe rust resistance (Fig. 2) Besides, LSD test showed significant differences among the subpopulations, justifying the use of the population structure in the panel in the GWAS model.

### Marker-trait association and annotation

#### Seedling stage resistance

**Fig. 3:**
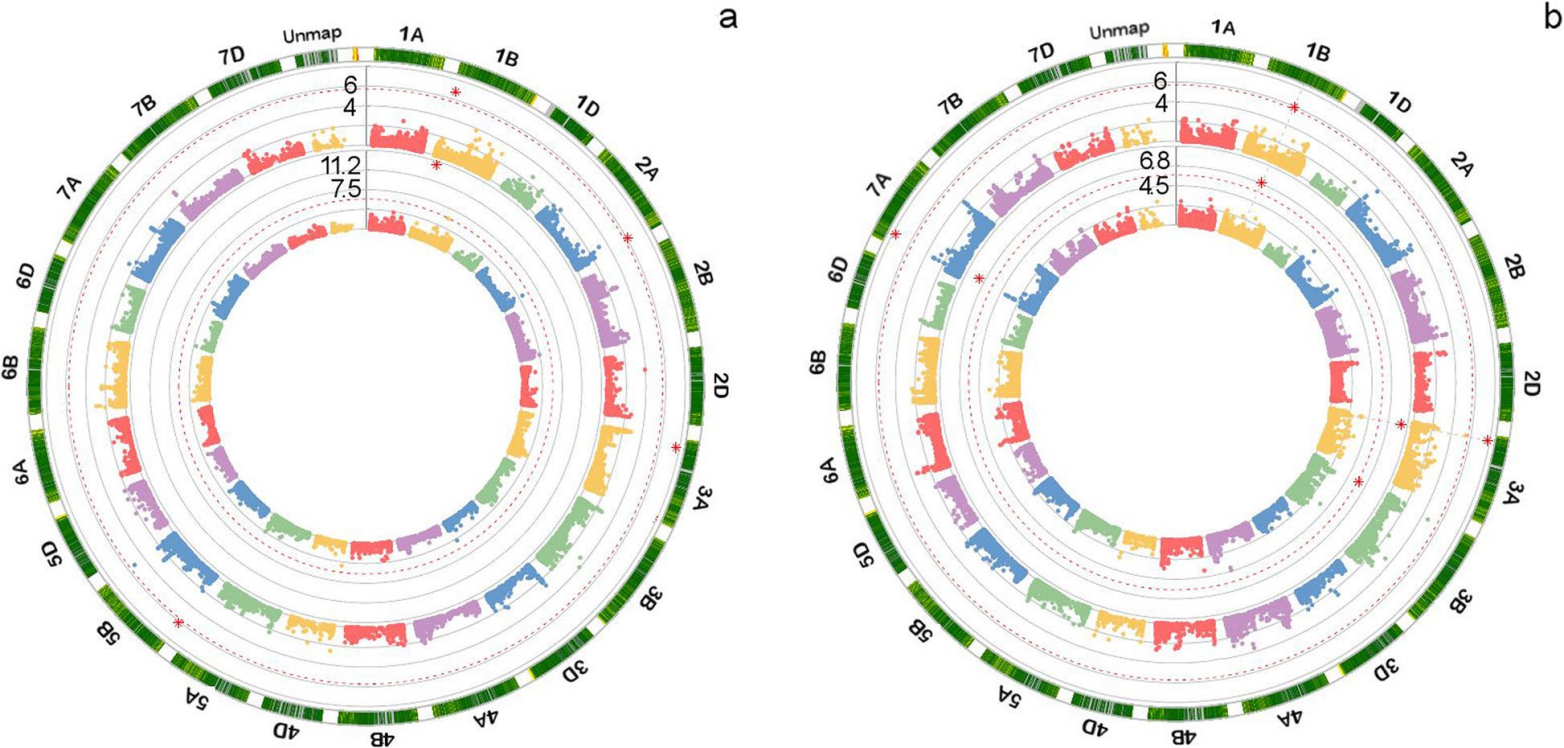
Circle Manhattan plots for stripe rust resistance in the collection of 245 spring bread wheat genotypes evaluated for **a** Seedling resistance (inside: infection type -IT- for the race *Yr19-71*; outside: IT for *Yr20-161*) and **b** Field-based resistance (inside: disease severity –DS-; outside: area under disease progress curve -AUDPC-). SNPs associated with stripe rust resistance are marked with red stars

**Fig. 4:**
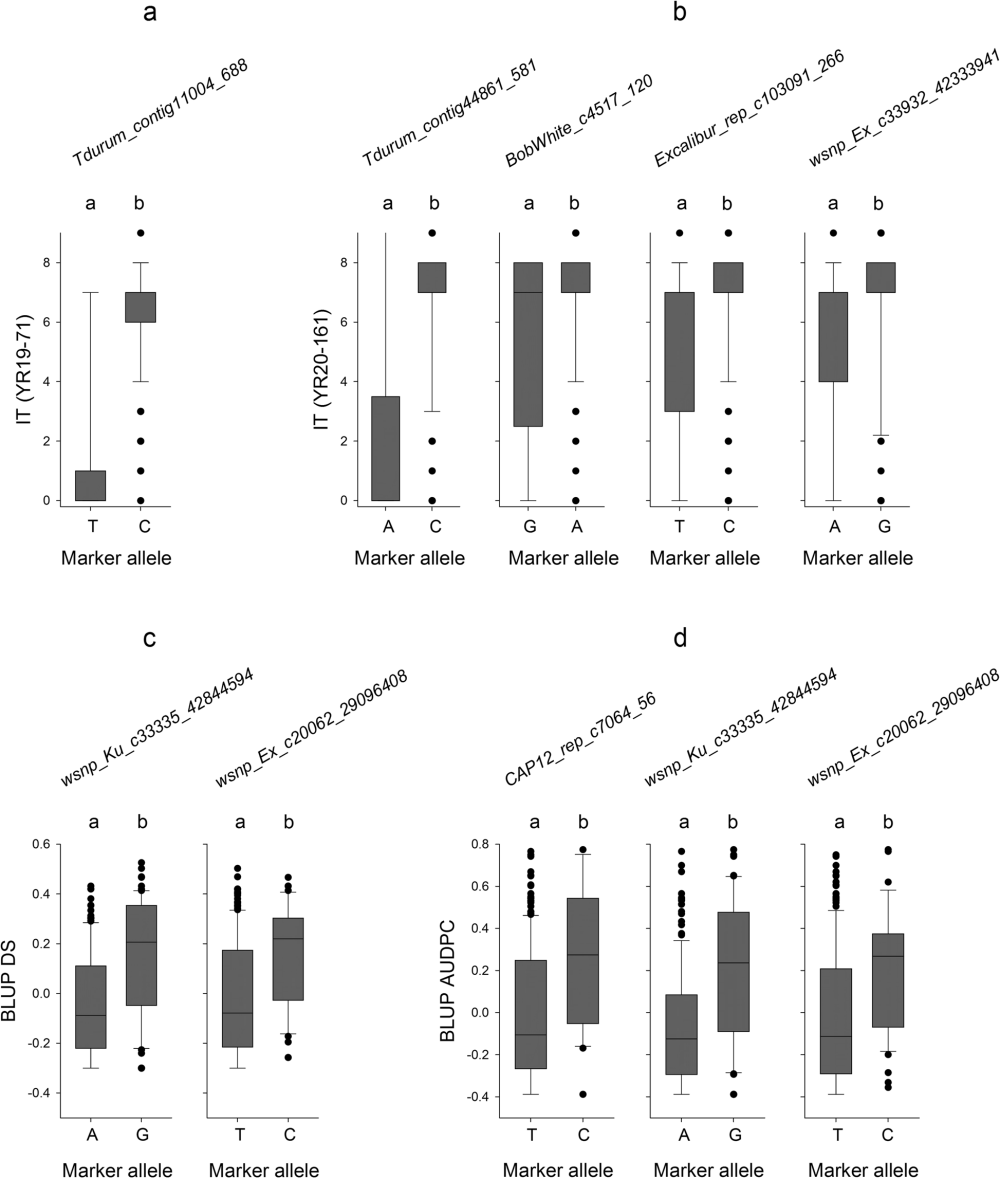
Allelic effects of the highly significant SNP markers on **a** BLUP-values of disease severity –DS- **b** BLUP-values of the Area Under Disease Progress Curve -AUDPC- **c** infection type -IT- for the race *Yr19-71* and **d** IT for *Yr20-161*. Left: Favorable (resistance-associated) allele; Right: unfavorable allele. The marker allele represented by the same lowercase letter(s) are not significantly different at *p* = 0.01

A total of five SNPs (at false discovery rate -FDR- adjusted *P* < 0.01) were significantly associated with the two races and were located on chromosomes 1B, 2A, 3A, and 5B (Table 2, Fig. 3a). The percentage of phenotypic variation (R^2^) for IT to *Yr19-71* explained by the significant marker, *Tdurum_contig11004_688*, located on chromosome 1B, was 32.6%, while the R^2^ for IT to *Yr20-161* ranged from 2% for *wsnp_Ex_c33932_42333941* on 5B to 16.5% for *Tdurum_contig44861_581* on 1B. The resistance alleles of the genomic regions showed allelic effects of reducing IT by 5.1 for *Yr19-71* and from 0.8 to 4.7 for *Yr20-161* (Fig. 4a and b). QQ-plots for IT to *Yr19-71* (Figure S1a) and *Yr20-161* (Figure S1b) reflected that the distribution of observed associations (*P*-values) was close to the distribution of the expected associations on the lower left section of the graph. SNPs on the upper right section, deviating from the diagonal, are most likely associated with these traits.

**Table 2:** Significant SNPs associated with seedling resistance to stripe rust in the collection of bread wheat

Trait	Chr.^a^	SNP	Position^b^	Allele ^c^	*P*-value	MAF ^d^	FDR-Adjusted *P*-values^e^	R^2f^
IT (*Yr19-71*)	1B	*Tdurum_contig11004_688*	62,487,859	C/T	5.02E^−15^	0.06	1.27 × 10^–10^	32.62
IT (*Yr20-161*)	1B	*Tdurum_contig44861_581*	28,019,260	C/A	1.59E_-07_	0.04	1.01 × 10^–03^	16.48
	2A	*BobWhite_c4517_120*	755,790,846	A/G	7.64E^−07^	0.35	3.23 × 10^–03^	6.59
	3A	*Excalibur_rep_c103091_266*	79,761,951	C/T	2.14E^−08^	0.32	3.29 × 10^–04^	8.34
	5B	*wsnp_Ex_c33932_42333941*	12,898,000	A/G	6.79E^−07^	0.33	3.23 × 10^–03^	1.99

^a^ *Chr.* Chromosome

^b^ Physical position of SNP (single-nucleotide polymorphism) markers in base pairs as per IWGSC Ref Seq 2.1

^c^ Favorable allele is underlined

^d^ *MAF* Minor allele frequency

^e^ F*DR* False discovery rate adjusted *P*-values

^f^ Percentage of phenotypic variation explained by the marker-trait association

#### Field-based resistance to stripe rust

A total of three SNPs, at FDR-adjusted *P* < 0.01, were associated with field-based resistance to stripe rust (Table 3, Fig. 3b), and they were located on chromosomes 3B and 7A. Of these, two SNPs were associated with DS, and all three were associated with AUDPC. Markers *wsnp_Ku_c33335_42844594 -*on chromosome 3B- and *wsnp_Ex_c20062_29096408* -on 7A- were commonly detected for the two traits. The phenotypic variation (R^2^) explained by the SNPs for DS was 13.4% for *wsnp_Ku_c33335_42844594* and 7.7% for *wsnp_Ex_c20062_29096408*; in turn, for AUDPC, R^2^ ranged from 7.8% for *wsnp_Ex_c20062_29096408*, to 14% for *wsnp_Ku_c33335_42844594*. The resistance alleles of the genomic regions showed allelic effects of reducing stripe rust responses ranging from 0.17 to 0.19 for best linear unbiased predictors (BLUP) of DS and 0.23 to 0.28 for BLUP of AUDPC (Fig. 4c and d). QQ-plots for DS (Figure S1c) and AUDPC (Figure S1d) reflected that the distribution of observed associations was close to the distribution of the expected associations on the lower left section of the graph and the SNPs on the upper right section of the graph, which deviate from the diagonal, are most likely associated with these traits.

**Table 3:** Significant markers associated with field-based resistance to stripe rust in the collection of bread wheat

Trait	Chr.^a^	SNP marker	Position^b^	Allele^c^	*P*-value	MAF ^d^	FDR-Adjusted *P*-values^e^	R^2f^
DS	3B	*wsnp_Ku_c33335_42844594*	71,871,162	A/G	2.26E-07	0.316	2.861 × 10^–03^	13.40
	7A	*wsnp_Ex_c20062_29096408*	96,809,079	T/C	8.10E-08	0.197	2.052 × 10^–03^	7.67
AUDPC	3B	*CAP12_rep_c7064_56*	43,970,547	T/C	1.47E-08	0.138	1.864 × 10^–04^	8.15
	3B	*wsnp_Ku_c33335_42844594*	71,871,162	A/G	4.06E-08	0.316	3.428 × 10^–04^	14.01
	7A	*wsnp_Ex_c20062_29096408*	96,809,079	T/C	5.81E-10	0.197	1.472 × 10^–05^	7.79

^a^ *Chr.* Chromosome

^b^ Physical position of SNP (single-nucleotide polymorphism) markers in base pairs as per IWGSC Ref Seq v2.1

^c^ Favorable allele is underlined

^d^ *MAF* Minor allele frequency

^e^ *FDR* False discovery rate adjusted *P*-values

^f^ Percentage of phenotypic variation explained by the marker-trait association

#### Relationship between the number of favorable alleles and response to stripe rust

**Fig. 5:**
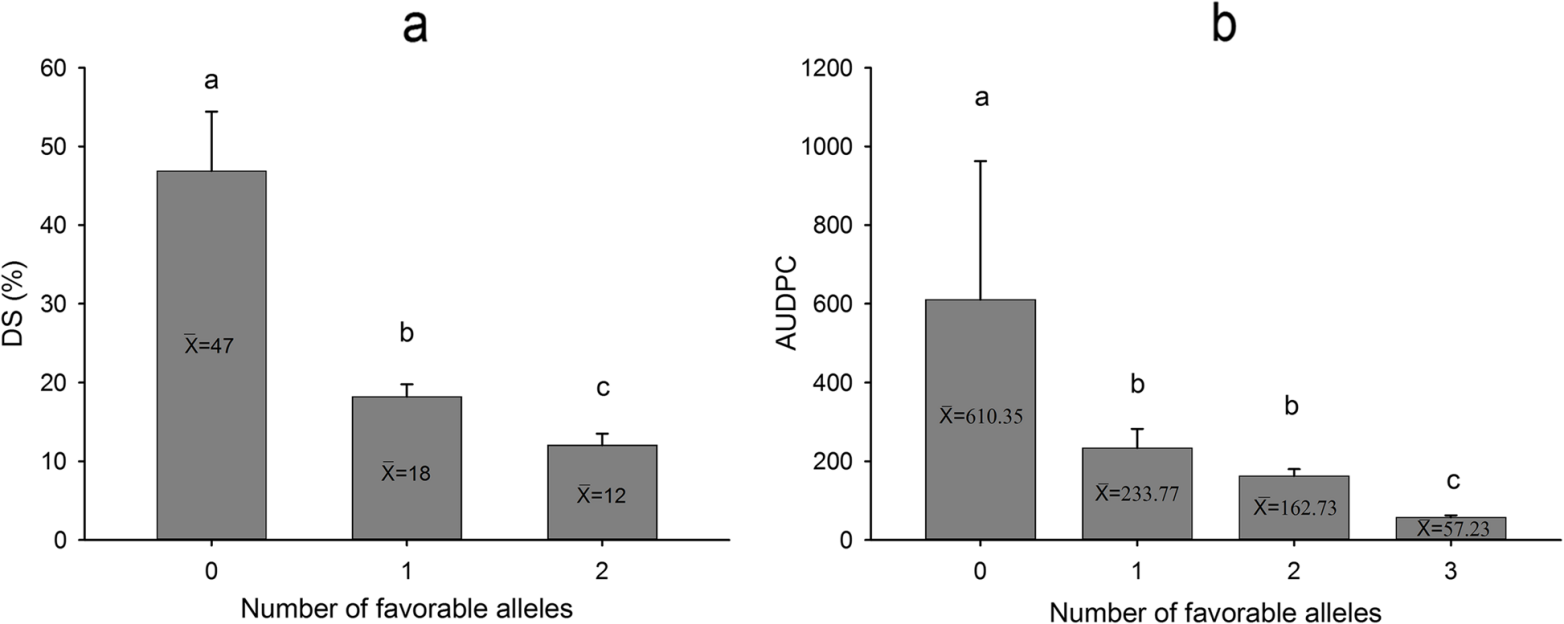
Effect of the number of resistant alleles on **a** DS and **b** AUDPC. The numbers in abscissa indicate number of favorable alleles in each subset of genotypes. The error bars indicate the mean standard error of each subset of genotypes. The subsets of genotypes represented by the same lowercase letter(s) are not significantly different at *p* = 0.01

After ranking the accessions by increasing order based on the number of APR-associated favorable alleles, a comparison between the groups evidenced a decrease in the average trait values in line with the increase in the number of resistance alleles (Fig. 5). For DS, genotypes that combined the two favorable alleles significantly improved APR to stripe rust reducing the mean by 35% compared with the genotypes with no such alleles (*P* = 1.41 × 10^–6^). In the same way, for AUDPC, accessions with the three resistance alleles significantly reduced the mean by 553.12 compared with the genotypes without any favorable allele (*P* = 2.4 × 10^–14^).

#### Comparison of *Pst* resistance QTL and *Yr* genes

**Fig. 6:**
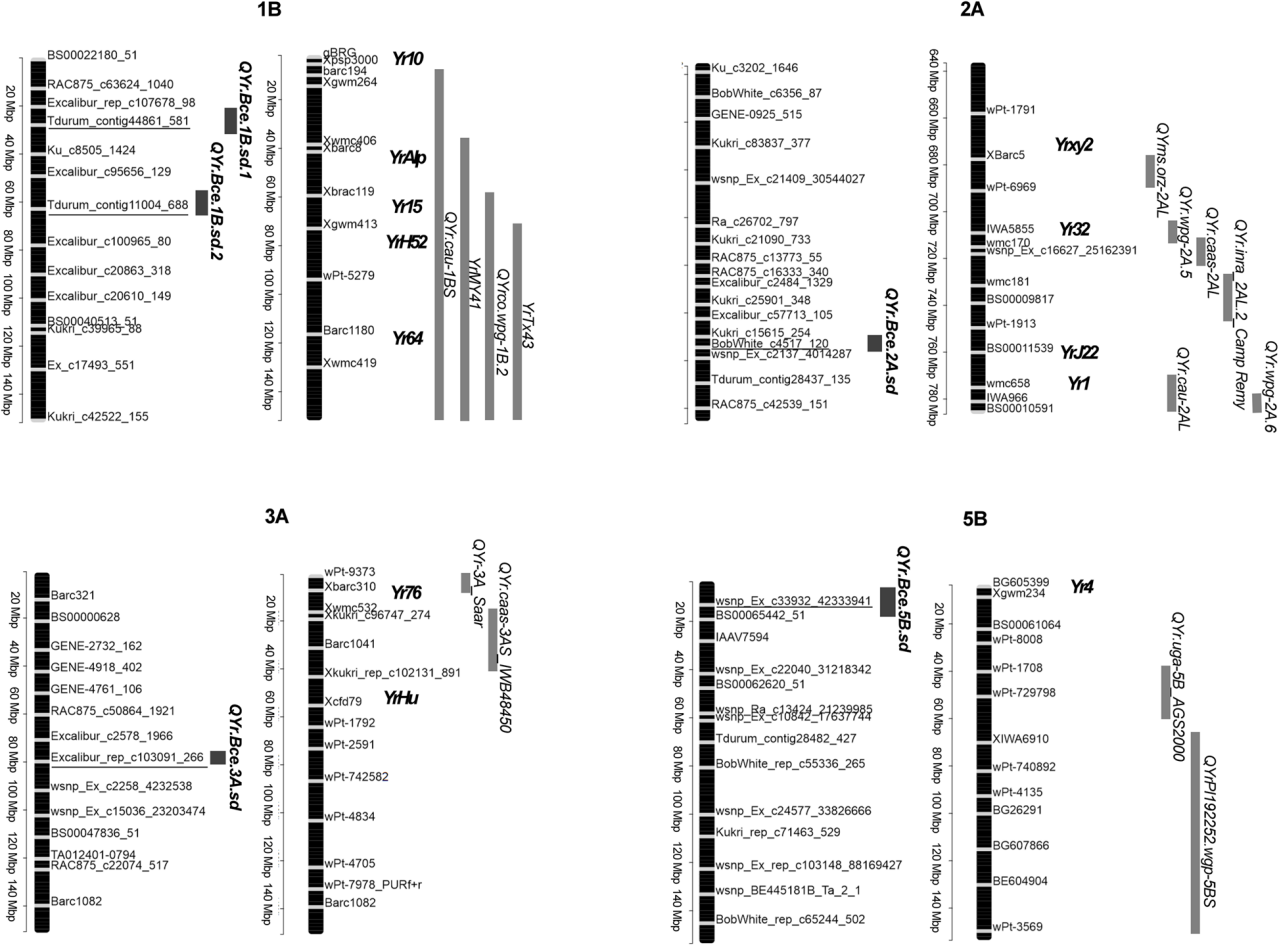
Comparisons of the chromosome positions (in Mb, denoted with horizontal grey marks on vertical black bars) between the QTL identified in this study associated with seedling resistance to stripe rust (left bar per chromosome) and the reported *Yr* genes and QTL (right bar per chromosome). SNP markers identified in this GWAS are underlined. All positions are approximate, and thus should be treated as guidelines for future studies. The detailed information of the previously mapped *Yr* genes and QTL is presented in Supplementary Table 6

**Fig. 7:**
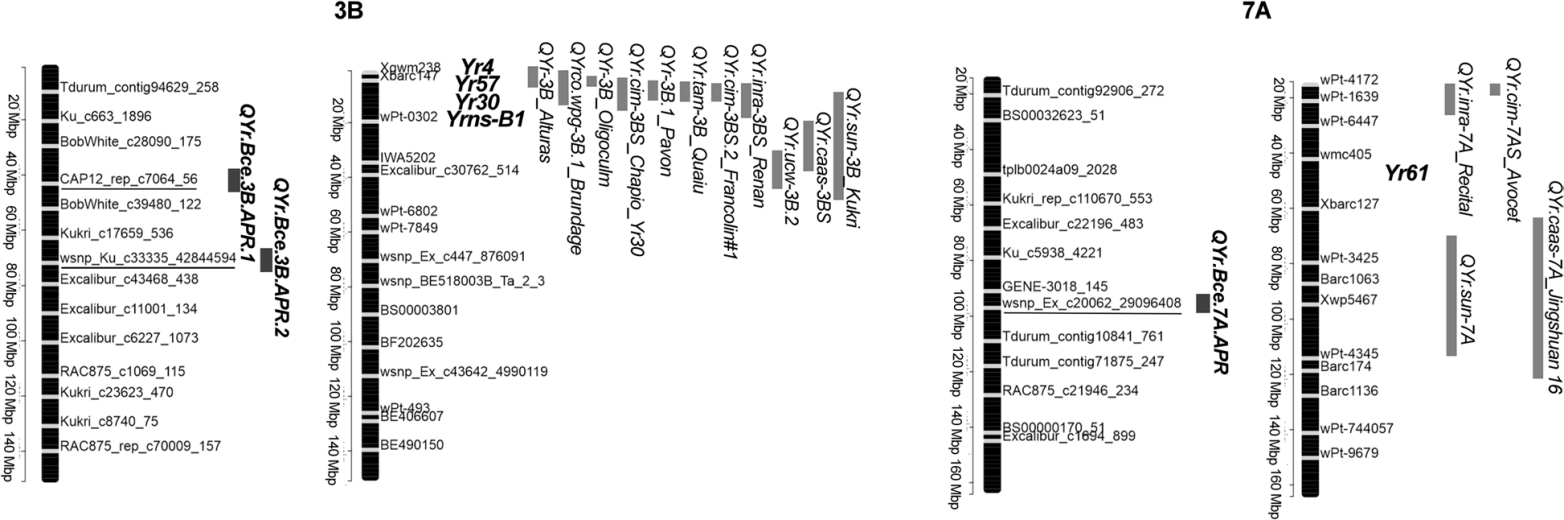
Comparisons of the chromosome positions (in Mb, denoted with horizontal grey marks on vertical black bars) between the QTL identified in this study associated with adult plant resistance to stripe rust (left bar per chromosome) and the reported *Yr* genes and QTL (right bar per chromosome). SNP markers identified in this GWAS are underlined. All positions are approximate, and thus should be treated as guidelines for future studies. The detailed information of the previously mapped *Yr* genes and QTL is presented in Supplementary Table 6

In order to identify which of the SNP markers described in Tables 2 and 3 mapped to similar regions to those of previously identified *Yr* resistance genes and QTL, physical positions of these markers were compared with the physical location of the flanking markers for each previously reported QTL or *Yr* gene [22, 23, 33, 44], according to the reference sequence of the bread wheat genome (Ref Seq v2.1; IWGSC) [46, 47]. QTL confidence intervals were established based on ± the intermarker physical distance corresponding to the critical LD r^2^ [22, 44](Fig S2). Five SNP markers on chromosomes 1B, 2A, 3B, 5B and 7A were mapped in the same region of previously reported stripe rust resistance QTL and/or genes. Other two SNPs on chromosomes 3A and 3B were mapped far from any currently known *Pst* resistance gene identified in *Triticum aestivum*. The remaining marker on chromosome 1B was mapped to the same region of a previously reported QTL, but it was determined to be different from this QTL based on the phenotype. Hence, these last three genomic regions most likely tag new *Pst* resistance loci. Figures 6 and 7 show the comparisons between the genomic regions reported here and the previously identified resistance genes and QTL mapped in the same regions. Detailed description of Figs. 6 and 7 are summarized in Table S4.

## Discussion

Evolution of new races of stripe rust in bread wheat is a recurrent threat for successful production of this crop in many regions around the world, including Argentina. Recently, the emergence of the new racial groups of *Pst*, caused a breakdown of many of the major resistance genes in germplasm adapted in the country [15, 16]. Therefore, the identification of new sources of resistance and their introgression into adapted germplasm is a pressing need for accelerating durable stripe rust resistance breeding in bread wheat.

### Response of spring wheat accessions to stripe rust

In this study, a large and diverse collection of spring wheat lines from several breeding programs was characterized for seedling resistance to two prevailing races of *Pst* in Argentina and field-based resistance during two years. Highly significant differences among the genotypes were found in both ASR and APR reflecting the effectiveness of the phenotyping strategy and the presence of a considerable genetic variation in the panel. Results from ASR evaluation showed that depending on the race, 15–17% of the genotypes were resistant while 67–75% displayed intermediate resistance and 8–10% were susceptible, indicating the presence of single major genes in the panel, which might include uncharacterized major *Yr* resistance genes.

On the other hand, among the 156 accessions that showed strong to moderate resistance in the field, with disease severity values < 0.2, 101 were resistant only at a post-seedling growth stage. This may indicate that the resistance in these genotypes is likely conferred by APR genes or QTL. The incorporation and deployment of these non-race-specific resistance genes is an important objective for wheat breeding programs because this kind of genes have historically provided more durable resistance than race-specific resistance genes [18, 30, 35].

The high degree of broad-sense heritability for DS and AUDPC (H^2^ = 0.89 and H^2^ = 0.93 respectively) indicates that the phenotypic variation in stripe rust resistance is stable and mainly explained by the genotypic variation. These values result favorable for the identification of significant associations in GWAS. Likewise, selection for highly resistant genotypes could be successful in future breeding programs. Similar broad-sense heritability values were reported in previous studies [22, 23].

Besides, highly significant differences were found between the years and genotype x year interaction indicating that the response of the genotypes differed in 2020 and 2021 (*P* < 0.0001). These statistically significant differences observed in the two years were most likely the result of variation in environmental variables (temperature and rainfall). In this situation, BLUP values were calculated to reduce the environmental impact and increase the reliability of the results. Nevertheless, a high degree of correlation was observed for *Pst* response between the two years (*r* = 0.82 for DS and *r* = 0.87 for AUDPC).

### Population structure of the spring bread wheat accessions

The power of association mapping studies depends on levels of genetic variation, linkage disequilibrium, and population structure [48]. Many studies have shown that the lack of appropriate correction for population structure can lead to false-positive trait-marker associations [49–52]. In this way, identifying and taking into consideration population structure is important before conducting GWAS to avoid spurious associations.

Population structure of the 245 accessions from the spring bread wheat collection was investigated by Zhang et al. [45] and the analysis revealed the clustering of the panel into four subpopulations as most likely representative. The characterization of the subgroups was mainly reflected by the geographic origin of the genotypes. In the present study, the population structure of the collection was used as a covariate in the GWAS to reduce the likelihood of false-positive associations. Additionally, a significant correlation was observed between the population sub-clusters and the response to stripe rust resistance, justifying the use of the population structure in the model.

### Significant associations in the GWAS

GWAS using high-quality SNP marker data and *Pst* assessments from field trials and greenhouse experiments provided valuable information for a comprehensive analysis of stripe rust resistance in the spring bread wheat collection. The recently developed BLINK method was used in the present study to explore single-marker associations. This last-generation GWAS algorithm, which implements a multiple *loci* test method by combining a fixed-effect model with Bayesian information criteria and uses linkage disequilibrium information, increases the statistical power while better controlling the false-positive rate [49]. QQ- plots reflected that the distribution of observed associations (*P*-values) was close to the distribution of the expected associations on the lower left section of the graph (Fig S1). This means the method implemented for GWAS was sufficiently stringent to control spurious associations.

A total of eight SNP markers were detected as being significantly associated with experiment-wise correction for multiple testing (FDR adjusted *P* < 0.01) in both seedling and field experiments. Analysis showed a significant effect due to pyramiding minor resistant alleles identified in this study. The accessions that harbored relatively few or none of the identified resistance-associated favorable alleles showed a comparatively high disease level. Similar to a previous report by Maccaferri et al. [22], Naruoka et al. [53], and Yao et al. [54], resistance to *Pst* was enhanced continuously with an increase in the number of favorable alleles, which revealed the additive effect of accumulation of alleles of field-based resistance QTL. This constitutes a promising result in the context of genetic improvement for durable resistance to *Pst*.

### Alignment of QTL to the previously identified Yr genes/QTL

The IWGSC genome assembly (IWGSC RefSeq v2.1) was used to compare resistance *loci* detected in the current study with previously mapped *Yr* genes and QTL. SNP markers on two genomic regions were mapped far from any previously identified *Yr* gene. Other marker was mapped on the same region of a previously reported QTL, but different based on the phenotype. Thus, these three genomic regions could tag new *Pst* resistance loci. The remaining genomic regions were mapped close to known *Yr* genes and QTL. Relationship of the significant genomic regions with previously mapped *Yr* genes and QTL are discussed below.

#### Chromosome 1B

To date, numerous *Pst* genes have been identified on chromosome 1B including: *Yr9* [55], *Yr10* [56], *Yr15* [57, 58], *Yr24/Yr26*, [56, 59] *Yr29* [60], *Yr64* [57] and *Yr65* [57]. Likewise, several temporarily designated *Yr* genes have been mapped to 1B including: *YrAlp* [61], *YrC142* [62], *YrCHK* [63], *YrExp1* [61] and *YrH52* [64] among others. Furthermore, many QTL associated with APR to stripe rust have been identified on this chromosome [44, 65, 66]. In this research, SNP *Tdurum_contig11004_688* (*QYr.Bce.1B.sd.2*)*,* identified on the short arm of chromosome 1B, was in association with the resistance to pathotype -*Yr19-71*.

Of the many *Yr* genes mapped to 1B, resistance gene *Yr15* is located in the same region as SNP *Tdurum_contig11004_688*. *Yr15* was discovered in the accession G25 of wild emmer wheat (*Triticum turgidum* ssp. *dicoccoides*), the tetraploid progenitor of hexaploid common wheat [67] and since then it has been introgressed into various tetraploid and hexaploid wheats. The *Yr15* gene was recently cloned by Klymiuk et al. [58] and has been described as a putative protein kinase-pseudokinase named *WTK1*, conferring all-stage resistance against more than 3000 genetically diverse *Pst* races, including modern races, such as ‘Warrior’. Although certain *Pst* races were reported to be virulent on *Yr15* [68], since 2003 there has not been informed virulence on *Yr15* in the newly isolated *Pst* races [69]. In accordance with this, the seedling test demonstrated that *Yr19-71* pathotype is avirulent on the *Yr15* gene. We know that the *Yr15* gene is present in some of the 245 spring bread wheat accessions of the collection used in this study. Therefore, it is likely that *Tdurum_contig11004_688* represents the *Yr15* gene.

#### Chromosome 2A

One genomic region (*QYr.Bce.2A.sd*) was identified on chromosome 2A in association with seedling resistance to *Yr20-161* pathotype. SNP *BobWhite_c4517_120* was found within the same chromosomal region where one temporarily designated gene *YrJ22* [70] is located. *YrJ22* is a dominant gene, identified in the Chinese wheat cultivar Jimai 22. For this gene we do not know the response to the *Yr19-71* and *Yr20-161* pathotypes since the source of *YrJ22* has not been tested against the two races. Further analyses are required to confirm whether the resistance conferred by SNP *BobWhite_c4517_120* is due to *YrJ22*.

#### Chromosome 3B

In the present study, *CAP12_rep_c7064_56* (*QYr.Bce.3B.APR.1*) was found to be associated with adult plant resistance to *Pst* on chromosome 3B. Previous studies have reported that numerous *Yr* genes and stripe rust resistance QTL are present on this chromosome. In the proximal end of the short arm of chromosome 3B, four *Yr* genes are present: *Yr4* [31], *Yr57* [71], *Yr30* [72] and the temporarily designated gene *Yrns-B1* [31]. Besides, in that chromosomal region a cluster of stripe rust APR QTL have been mapped: *QYr-3B_Alturas*, a high-temperature adult-plant resistance QTL identified in the American spring bread wheat cultivar Alturas [73]; *QYrco.wpg-3B.1_Brundage* identified in the winter bread wheat cultivar Brundage [66]; *QYr-3B_Oligoculm* in the Israeli bread wheat Oligoculm [72]; *QYr.cim‐3BS_Chapio* in the spring bread wheat cultivar Chapio [74]; *QYr-3B.1_Pavon* in the spring bread wheat cultivar Pavon76 [75]; *QYr.tam-3B_Quaiu* in bread wheat cultivar Quaiu 3 [76]; *Yr.cim-3BS.2_Francolin#1* identified in the cultivar Francolin#1 [77]; *QYr.inra-3BS_Renan* in cultivar Renan [78]; *QYr.ucw-3B.2* identified in a worldwide collection of spring bread wheat [22]; *QYr.caas-3BS* in the Chinese winter wheat cultivar Zhong 892 [79] and *QYr.sun-3B_Kukri*, identified in the cultivar Kukri [80]. Marker *CAP12_rep_c7064_56* in our study was mapped within the confidence interval of the *QYr.sun-3B_Kukri*. Therefore, it is likely that resistance conferred by *CAP12_rep_c7064_56* is linked to the QTL *QYr.sun-3B_Kukri* identified by Bariana et al. [80].

#### Chromosome 5B

One genomic region (*QYr.Bce.5B.sd*) was identified on chromosome 5B in the same chromosomal region of the designated gene for resistance to stripe rust *Yr47* [81]. *Yr47* is a seeding resistance gene identified in Iranian common wheat landraces [81]. However, it is not known the response of *Yr47* to the *Yr19-71* and *Yr20-161* pathotypes. As the source of *Yr47* has not been tested against the two pathotypes used in this study, further analysis may be required for clarification.

#### Chromosome 7A

SNP *wsnp_Ex_c20062_29096408* was found to be associated with APR to *Pst* (both DS and AUDPC) on chromosome 7A (*QYr.Bce.7A.APR*). In this chromosomal region two QTL: *QYr.sun-7A* identified in the synthetic hexaploid CPI133872 [82] and *QYr.caas-7A_Jingshuan* 16 mapped in the Chinese wheat cultivar Jingshuan 16 [83] have been reported. *QYr.sun-7A* and *QYr.caas-7A_Jingshuan*.16, which were mapped close to *wsnp_Ex_c20062_29096408*, were reported as minor APR QTL; therefore, it is likely that the marker identified in this study is linked to these QTL. The identity or similarity of the genomic region tagged by *wsnp_Ex_c20062_29096408* with previously mapped QTL needs to be further investigated.

### Novel potential genomic regions identified in this study and their significance

Three genomic regions identified in this study might be tagging novel genomic regions. Two of these regions on chromosomes 3A (*QYr.Bce.3A.sd*) and 3B (*QYr.Bce.3B.APR.2*) were mapped where no *Pst* genes or QTL have been previously reported. The third region (*QYr.Bce.1B.sd.1*) was in the same location of a previously reported QTL, but was determined to be different from this QTL based on the phenotype.

SNP *Tdurum_contig44861_581* (*QYr.Bce.1B.sd.1*) was mapped in the proximal end of the short arm of chromosome 1B between two previously reported genes: *Yr10* [84] and *YrAlp* [61]. *Yr10* gene was mapped in association with the *Xpsp3000* marker at 5.15 Mb, *whereas Tdurum_contig44861_581* detected in this study is located at 28 Mb. Likewise *YrAlp* was associated with the *Xwgp47* marker at 58.7 Mb. On the other hand, SNP *Tdurum_contig44861_581* overlapped with the previously reported QTL *QYr.cau-1BS* [82]. However, the marker identified in this study confers seedling resistance of low IT, and *QYr.cau-1BS* is not associated with the seedling resistance (exhibits high/susceptible IT), but it is associated with the adult plant resistance (latent period). These findings indicate that *QYr.Bce.1B.sd.1* could be a novel *Pst* resistance gene.

One genomic region (*QYr.Bce.3A.sd*) was identified on chromosome 3A in association with seedling resistance to the *Yr20-161* race. Stripe rust seedling resistance SNP *Excalibur_rep_c103091_266* was identified in the short arm of chromosome 3A where two previously reported *Yr* genes were mapped: *Yr76* gene, identified in the winter club wheat cultivar Tyee [85] and *YrHu* [86] identified in *Psathyrostachys huashanica,* a related species to *Triticum aestivum*. Nevertheless, *QYr.Bce.3A.sd* did not map within the confidence interval of these genes. Hence *QYr.Bce.3A.sd* might be a new stripe rust resistance gene.

The marker *wsnp_Ku_c33335_42844594*, designated as *QYr.Bce.3B.APR.2*, was found in association with seedling resistance against the *Yr20-161* pathotype in the short arm of chromosome 3B. Four *Yr* resistance genes *Yr4* [31], *Yr57* [31], *Yr30* [72] and *Yrns-B1* [31] are reported on this region. However, according to the reference sequence -Ref Seq v2.1- of the IWGSC, *wsnp_Ku_c33335_42844594* is outside the genomic region of any of these reported genes. While the marker identified here is mapped at 71.87 Mb, *Yr4*, *Yr57*, *Yr30* and *Yrns-B1* are located at 4.9, 4.94, 6.7 and 17.6 Mb respectively. Thus, based on its physical position, *QYr.Bce.3B.APR.2* could be representing a potential new source for resistance to stripe rust.

The results presented here, give an overview of the relationships between the loci identified in this study and the previously identified *Pst* resistance genes and QTL. Nevertheless, these results should be considered with prudence because of the intrinsic limitations of published maps, which can result in distorted distances in some region of the map. Besides, the low resolution of the original maps of *Pst* genes and the extended LD in wheat make the comparisons are difficult. In this way, the relationships described in this section should be considered as tentative.

## Conclusion

The results of the present study highlight the possibility of exploiting the high genetic diversity in wheat germplasm collections to identify genomic regions associated with the resistance to stripe rust. The collection of spring bread wheat exhibited a wide range of phenotypic variation for both field-based and seedling resistance to stripe rust. Genotypes with a higher percentage of alleles associated with the stripe rust resistance constitute valuable genetic resources that could be used as parents in Argentine breeding programs to improve stripe rust resistance. In the present study, we identified eight QTL associated with the resistance to stripe rust; three of them could be probably novel. The markers linked to QTL identified in the current research result of substantial value for marker-assisted selection in wheat breeding programs. These genomic regions constitute the initial step to search for their candidate genes, which will allow their better manipulation in the future

## Methods

### Plant material

A collection of 245 spring bread wheat (*Triticum aestivum* L.) genotypes, already described in Zhang et al. [45] and well adapted to Argentinian environments, was used in this study. Briefly, the panel from several breeding programs, included 69 genotypes from CIMMYT (CMT), 12 from South Dakota State University (SDK), 42 from the University of California, Davis (UCD), 26 from the University of Idaho (UIA), 19 from the University of Minnesota (UMN), 15 from Washington State University (WAS), and 62 from various locations (other US programs and 14 other countries, Table S5).

As described by Zhang et al. [45], genotyping was carried out at the USDA-ARS genotyping laboratory, Fargo, ND using the Infinium wheat SNP 90 K iSelect assay (Illumina Inc., San Diego, CA, USA) developed by the International Wheat SNP Consortium [85]. After removing those SNPs with very low minor allele frequency (MAF) < 3% and/or > 10% missing values, a total of 22,226 high-quality SNP markers were used for the GWAS.

Population structure was investigated by Zhang et al. [45] using STRUCTURE 2.3.4 [48] and principal component analysis (PCA) with the R package *ade4* (Fig. S1) [87]. In short, four subpopulations were determined as most likely representative of the population structure, and the corresponding Q-matrices (4 × 245) of population membership coefficients were obtained (Table S5). The extent of linkage disequilibrium (LD) in this association panel was calculated by Zhang et al. [45], based on pairwise LD squared correlation coefficients (r^2^) for all intra-chromosomal SNP *loci*. The scatter plot of r^2^ versus physical distance was fitted using a nonlinear model described by Remington et al. [88] in R [89] with the function *nls* (nonlinear least squares method). The physical distance at which LD fell below the r^2^ thresholds -determined by Zhang, et al. [45]- was used to define the confidence intervals of the QTL detected in this study.

Additionally, a group of 24 genotypes was used in this work as differential testers of pathogenic variability of *Pst* (Table S6). The group included a set of 19 near-isogenic lines (NILs) developed by Dr. Wellings from Sydney University, three genotypes carrying specific gene or gene combinations kindly provided by CIMMYT, Mexico, and two European cultivars. The NILs are based on the Australian cultivar Avocet (of a high degree of susceptibility to *Pst*) and represent a set of wheat lines that are similar except for the presence of single genes for resistance. Furthermore, two Argentinian cultivars were included as checks in the experiments: Don Mario Algarrobo (universally susceptible to *Pst*) and Baguette 750 (susceptible to races of *Pst* with a higher virulence spectrum).

Finally, three highly susceptible local cultivars to the main races of *Pst* detected in the wheat region: Buck Claraz, SN 90, and Klein Lanza, were included as checks in the field experiments. These cultivars were chosen for presenting average severity values above 0.7 in the wheat region during the last years.

### Phenotypic trait evaluation

#### Seedling resistance screening

Seedling resistance to stripe rust was characterized under controlled conditions in a greenhouse at the INTA Bordenave Experimental Station (37°45′45’’ S; 63°05′28’’ W; 205 m.a.s.l.), Buenos Aires province, Argentina. Races *Yr19-71* and *Yr20-161*, representing the most prevalent races of the most frequent genetic groups of *Pst* in Argentina were used [90, 91]. Ten seeds of each genotype of the population were grown in plastic pots filled with a potting medium. When the two first leaves were fully expanded, the seedlings were inoculated by spraying the *Yr19-71* / *Yr20-161* urediniospores suspended in mineral oil (Soltrol 170) using an atomizer. Inoculated seedlings were sprayed with a fine mist of sterile water and incubated at 8 °C for 18 h in a dew chamber with relative humidity close to 100%. Then, seedlings were transferred to a greenhouse with a mean temperature of 16 ± 2 °C. Infection type (IT) was recorded for each seedling 15 days after inoculation based on a 0–9 scale [92]. Lines of the stripe rust differential set were also characterized for IT response to these two races in order to obtain the virulence/avirulence formulae of the races (Table S7).

#### Field-based resistance screening

Adult plants of the 245 genotypes were evaluated for response to *Pst* natural infection in field experiments at the INTA Balcarce Experimental Station (37°46′01’’ S; 58°18′29’’ W; 118 m.a.s.l.), Buenos Aires province, Argentina in two crop seasons (2020 and 2021). Sowing dates were August 4^th^ in 2020 and August 6^th^ in 2021. In the two field experiments, a non-replicated augmented design with eight blocks was used. Plots consisted of a single 1 m-long row with 30 cm between rows. Sowing density was adjusted to 350 plants m^2^. The susceptible checks Buck Claraz, SN 90, and Klein Lanza, were replicated twice in each block. The same checks also were planted as spreader rows bordering the trials to provide uniform stripe rust infection across the plots. Experiments were rainfed and conducted under optimal nutritional conditions.

Disease assessment started when most flag leaves of the susceptible checks displayed a disease severity of at least 50% and continued at least four times at 4-day intervals. Disease severity (DS) was visually assessed, based on the percentage of leaf area covered with uredinia, according to the modified Cobb’s scale [93]. After the last DS score when the disease progress ended, the area under the disease progress curve (AUDPC) was calculated according to the method used by Wilcoxon et al. [94].

#### Statistical analyses of the phenotypic data

Statistical analyses were performed using R software [89]. The IT data were analyzed by fitting linear models with the *lm* function. The statistical model used was:

$${y}_{ij} = {\mu }_{i}+{\varepsilon }_{ij}$$

where ${y}_{ij}$ is the response variable of genotype “*i*” on repetition “*j*”, $\mu$ is the mean value of the response variable of genotype “*i*”, and ${\varepsilon }_{ij}$ is the random error of the observation of genotype “*i*” on repetition “*j*”.

Assumptions on this model are: ${y}_{ij }\sim N\left(\mu ; {\sigma }_{e}^{2}\right)$ and ${\varepsilon }_{ij }\sim N\left(0; {\sigma }_{e}^{2}\right)$. ANOVA was performed to determine the significance of the effects of genotypes.

DS and AUDPC data were analyzed by fitting linear mixed models with the *lme* function from package *nlme* [95]. Models were fitted considering years and blocks as fixed factors and genotypes and genotype x year interaction as random factors. The statistical model used was:

$${y}_{ikj} = \mu +{\alpha }_{j}+{\beta }_{k\left(j\right)}{+ \tau }_{i}+{\gamma }_{j\left(i\right)}+{\varepsilon }_{ijk}$$

where ${y}_{ijk}$ is the response variable of genotype “*i*” on block “*k*” in the year “*j*”, $\mu$ is the mean value of the response variable,${\alpha }_{j}$ is the fixed effect of the year “*j*”, ${\beta }_{k\left(j\right)}$ is the fixed effect of the block “*k*” in the year “*j*”, ${\tau }_{i}$ is the random effect of genotype “*i*”, ${\gamma }_{j\left(i\right)}$ is the random interaction effect between genotype “*i*” and year “*j*”, and ${\varepsilon }_{ijk\left(s\right)}$ is the random error of the observation of genotype “*i*” on repetition “*k*” in the year “*j*”.

Assumptions on this model are: ${\alpha }_{j}\sim N\left(0; {\sigma }_{g}^{2}\right), { \beta }_{k\left(j\right)}\sim N\left(0; {\sigma }_{g}^{2}\right), {\tau }_{i} \sim N\left(0; {\sigma }_{g}^{2}\right)$, ${\gamma }_{j\left(i\right)}\sim N\left(0; {\sigma }_{ge}^{2}\right)$ and ${\varepsilon }_{ijk}\sim N\left(0; {\sigma }_{res}^{2}\right),$ all are independent of each other.

Sequential restricted maximum likelihood ratio tests were performed to determine the significance of the random effects of genotypes and genotype by year interactions. Best linear unbiased predictors (BLUPs) were obtained for all the genotypes. Variance components for genotypes, years, and genotype by year interactions were estimated using the Restricted Maximum Likelihood (REML) method [96]. Broad-sense heritabilities (H^2^) were estimated from variance components according to Hallauer et al. [97]. Pearson correlation coefficients were calculated between the subpopulations and response to stripe rust resistance. Least significant differences (LSD) test was performed among the subpopulation according to the response to stripe rust resistance.

#### Association analysis

To identify *loci* associated with responses to stripe rust, GWAS was performed using 22,226 informative SNPs in the set of 245 genotypes. Association analyses were implemented in GAPIT 3 (Genomic Association and Prediction Integrated Tool v3) (Gapit 2021; [98]) in R software [89]. Association analyses were performed using Bayesian- information and linkage-disequilibrium iteratively nested keyway -BLINK, [99]-. The population structure was used as covariate to control for spurious associations. The quantile–quantile (QQ) plot is a useful tool for assessing how well the model used in GWAS accounts for population structure. The majority of the points in the QQ-plot should lie on the diagonal line. Deviations from this line suggest the presence of spurious associations due to population structure and familial relatedness. It is expected that the SNPs on the upper right section of the graph deviate from the diagonal and these SNPs are most likely associated with the trait under study [98].

In order to minimize the chance of false-positive marker-trait associations, significant association *loci* were considered at genome-wide adjusted *P* < 0.01 based on the False Discovery Rate (FDR) multiple correction method [100]. Linear models were performed to assess the amount of phenotypic variation explained by the significant markers. The markers were used as the independent variable and BLUP of DS and AUDPC and IT values were included as the response variable. Circular Manhattan plots were generated by GAPIT 3 using R packages *gplot* and *scatterplot3d*. Names assigned to the QTL identified in this study start with the prefix “Q” for QTL, followed by “Yr” for yellow rust, “Bce” for Balcarce, chromosome name and “sd” for seedling trait or “APR” for adult plant resistance trait.

To assess the pyramiding effect of resistant alleles of QTL for APR identified in this study, the genotypes were classified into groups containing different numbers of resistant alleles. Differences between DS and AUDPC of these groups were compared using the Least Significant Differences (LSD) Test.

#### Comparison of *Pst* resistance QTL and *Yr* genes

In order to determine whether the significant SNPs detected in this study were located in the same position as that of previously reported *Yr* genes and resistance QTL, the physical locations of the genomic regions were compared. The physical positions were determined according to the reference sequence -Ref Seq v2.1- of the International Wheat Genome Sequencing Consortium (IWGSC) [46]. For the segments of the chromosomes containing the genomic regions detected in this research, previously reported *Yr* genes and resistance QTL were integrated in a map including different marker types. Genomic regions were drawn with chromoMap in R [101] Information of these *Yr* genes and QTL are presented in Table S4.

## Supplementary Information

**Additional file 1: Supplementary Table S1. **Analysis of variance (ANOVA) to determine the significance of the fixed effects of genotypes for Infection type for *Yr19-71* and *Yr20-161* races.** Supplementary Table S2.** Sequential restricted maximum likelihood ratio tests to determine the significances of the random effects of genotypes and genotype by year interactions for DS and AUDPC. **Supplementary Table S3.** Variance component estimates (genotypic, genotype x year interaction and residual variances) and broad-sense heritability (H^2^) for the analyzed traits. **Supplementary Table S4.** Information of the reported stripe rust resistance genes or quantitative trait loci on the segments of the chromosomes containing the genomic regions identified in this study. **Supplementary Table S5. **Accession number, synonyms, origin, pedigrees and subpopulation for the 245 bread wheat accessions included in the GWAS. **Supplementary Table S6.** Wheat differential lines used for race typing of *Puccinia striiformis* f. sp. *tritici*. **Supplementary Table S7.** Virulence/avirulence formula of the *Puccinia striiformis* f. sp. *tritici* races used for seedling resistance screening.

**Additional file 2: Supplementary Figure S1. **Structure analysis in the spring wheat association-mapping panel. a) The STRUCTURE analysis showed four hypothetical subpopulations represented by different colors. b) First two components (PC1 and PC2) of a principal component analysis of the spring wheat accessions color coded by breeding program (adapted from Zhang, et al. [45]). c) Neighbor-joining phylogenetic tree showing the subpopulations corresponding to the structure analysis. **Supplementary Figure S2. **Quantile-quantile (QQ) plots of the observed and the expected p values of the GWAS model. a) QQ-plot for seedling resistance (infection type -IT- for the races *Yr19-71* and *Yr20-161*); b) QQ-plot for Adult plant resistance (Disease Severity and area under disease progress curve –AUDPC-). **Supplementary Figure S3.** Linkage disequilibrium (LD) decay over physical distance. The scatter plots showing pairwise SNP markers LD r^2^ value as a function of inter-marker physical distances (Mbp) of (a) 1B chromosome; (b) 2A chromosome; (c) 3A chromosome; (d) 3B chromosome; (e) 5B chromosome; (f) 7A chromosome. The red curve represents the model fit to LD decay. The blue dashed line represents the specific critical r^2^ value beyond which LD is likely due to linkage. The green dashed line represents the confidence interval for the quantitative trait loci regions in which LD r^2^ = critical r^2^ value.

## Data Availability

The datasets used and/or analyzed during the current study are available from the corresponding author on reasonable request.

## Abbreviations

APR: Adult plant resistance
ASR: All-stage resistance
AUDPC: Area under disease progress curve
BLINK: Bayesian- information and linkage-disequilibrium iteratively nested keyway
BLUPs: Best linear unbiased predictors
DS: Disease severity
FDR: False Discovery Rate
GWAS: Genome-wide association study
H^2^: Broad-sense heritability
IT: Infection type
IWGSC: International Wheat Genome Sequencing Consortium
LD: Linkage disequilibrium
LSD: Least Significant Differences
PCA: Principal component analysis
*Pst*: *Puccinia striiformis* f. sp. *tritici*
QTL: Quantitative Trait locus
REML: Restricted maximum likelihood ratio
SNP: Single nucleotide polymorphism
*Yr*: Yellow rust

## Declarations

### Methodological statement

All methods were carried out in accordance with the appropriate guideline for plant material used in the study.
