# Supplementary figures and images for: Genome-wide association study for resistance in bread wheat (Triticum aestivum L.) to stripe rust (Puccinia striiformis f. sp. tritici) races in Argentina
Source: BMC Plant Biol. 2022 Nov 24;22:543. doi: 10.1186/s12870-022-03916-y (PMC9701071; doi:10.1186/s12870-022-03916-y)

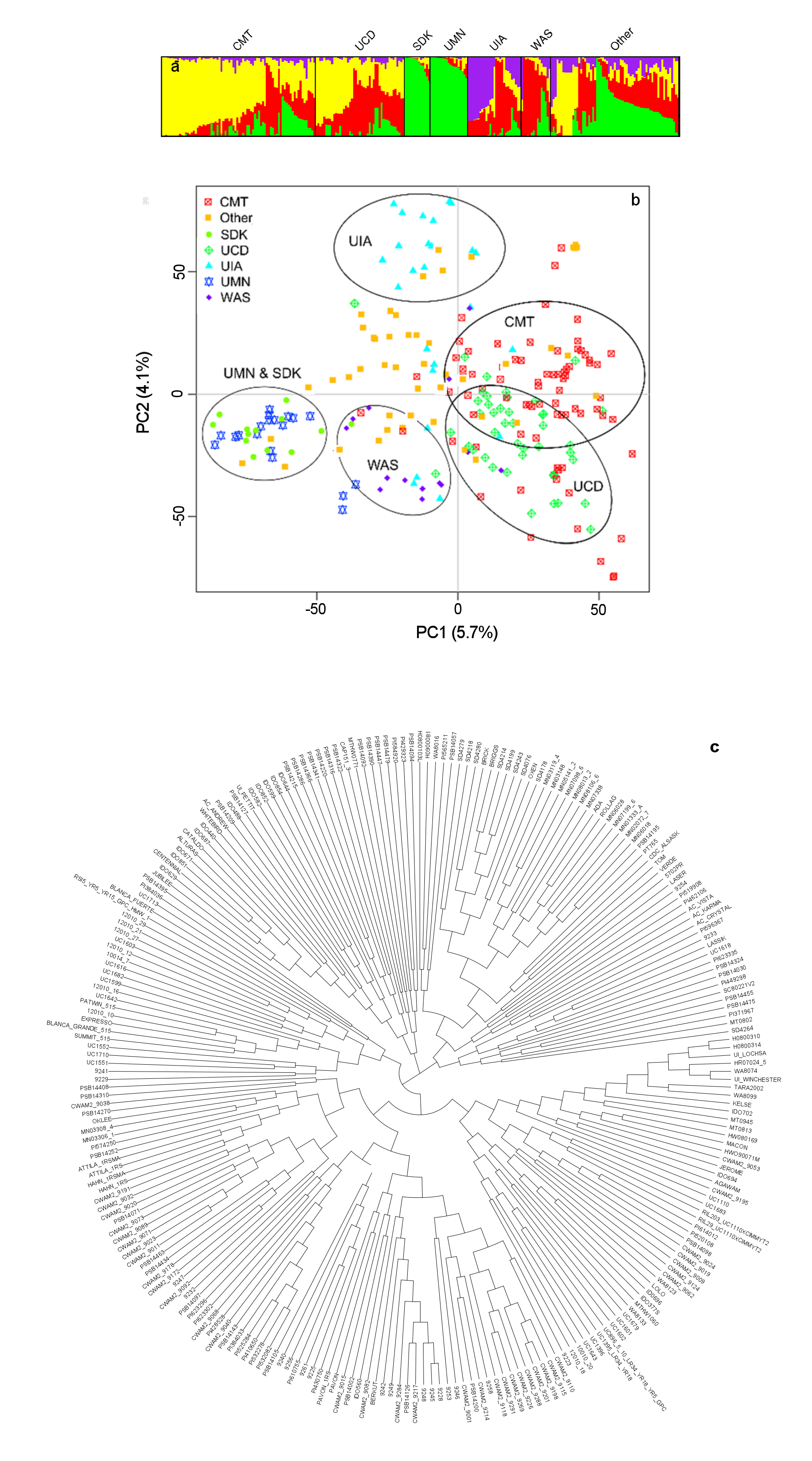

Supplement: Supplementary file 2 — Additional file 2: Supplementary Figure S1. Structure analysis in the spring wheat association-mapping panel. a) The STRUCTURE analysis showed four hypothetical subpopulations represented by different colors. b) First two components (PC1 and PC2) of a principal component analysis of the spring wheat accessions color coded by breeding program (adapted from Zhang, et al. [45]). c) Neighbor-joining phylogenetic tree showing the subpopulations corresponding to the structure analysis. Supplementary Figure S2. Quantile-quantile (QQ) plots of the observed and the expected p values of the GWAS model. a) QQ-plot for seedling resistance (infection type -IT- for the races Yr19-71 and Yr20-161); b) QQ-plot for Adult plant resistance (Disease Severity and area under disease progress curve –AUDPC-). Supplementary Figure S3. Linkage disequilibrium (LD) decay over physical distance. The scatter plots showing pairwise SNP markers LD r2 value as a function of inter-marker physical distances (Mbp) of (a) 1B chromosome; (b) 2A chromosome; (c) 3A chromosome; (d) 3B chromosome; (e) 5B chromosome; (f) 7A chromosome. The red curve represents the model fit to LD decay. The blue dashed line represents the specific critical r2 value beyond which LD is likely due to linkage. The green dashed line represents the confidence interval for the quantitative trait loci regions in which LD r2 = critical r2 value. [file 12870_2022_3916_MOESM2_ESM.zip › Additional file 2 Supplementary Figure S1.png]

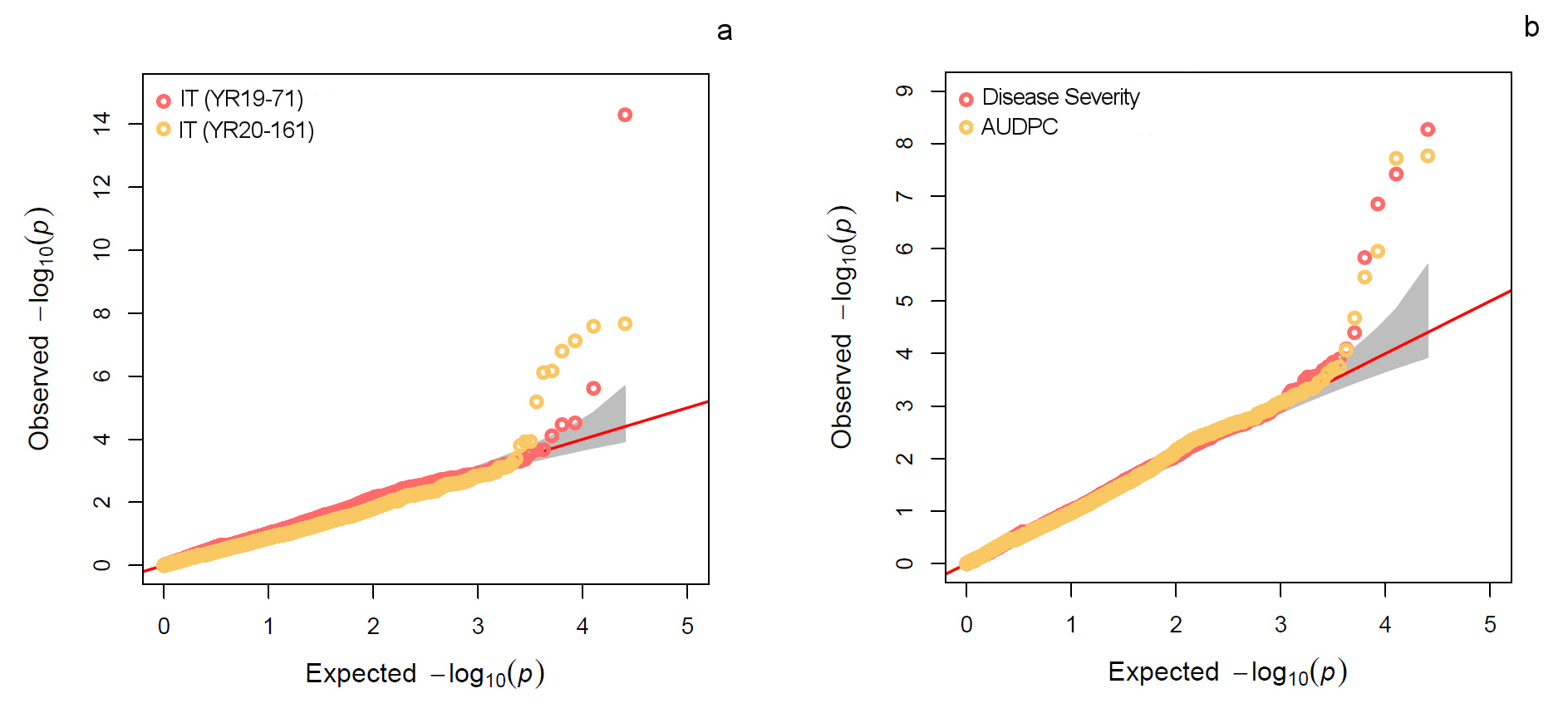

Supplement: Supplementary file 2 — Additional file 2: Supplementary Figure S1. Structure analysis in the spring wheat association-mapping panel. a) The STRUCTURE analysis showed four hypothetical subpopulations represented by different colors. b) First two components (PC1 and PC2) of a principal component analysis of the spring wheat accessions color coded by breeding program (adapted from Zhang, et al. [45]). c) Neighbor-joining phylogenetic tree showing the subpopulations corresponding to the structure analysis. Supplementary Figure S2. Quantile-quantile (QQ) plots of the observed and the expected p values of the GWAS model. a) QQ-plot for seedling resistance (infection type -IT- for the races Yr19-71 and Yr20-161); b) QQ-plot for Adult plant resistance (Disease Severity and area under disease progress curve –AUDPC-). Supplementary Figure S3. Linkage disequilibrium (LD) decay over physical distance. The scatter plots showing pairwise SNP markers LD r2 value as a function of inter-marker physical distances (Mbp) of (a) 1B chromosome; (b) 2A chromosome; (c) 3A chromosome; (d) 3B chromosome; (e) 5B chromosome; (f) 7A chromosome. The red curve represents the model fit to LD decay. The blue dashed line represents the specific critical r2 value beyond which LD is likely due to linkage. The green dashed line represents the confidence interval for the quantitative trait loci regions in which LD r2 = critical r2 value. [file 12870_2022_3916_MOESM2_ESM.zip › Additional file 2 Supplementary Figure S2.png]

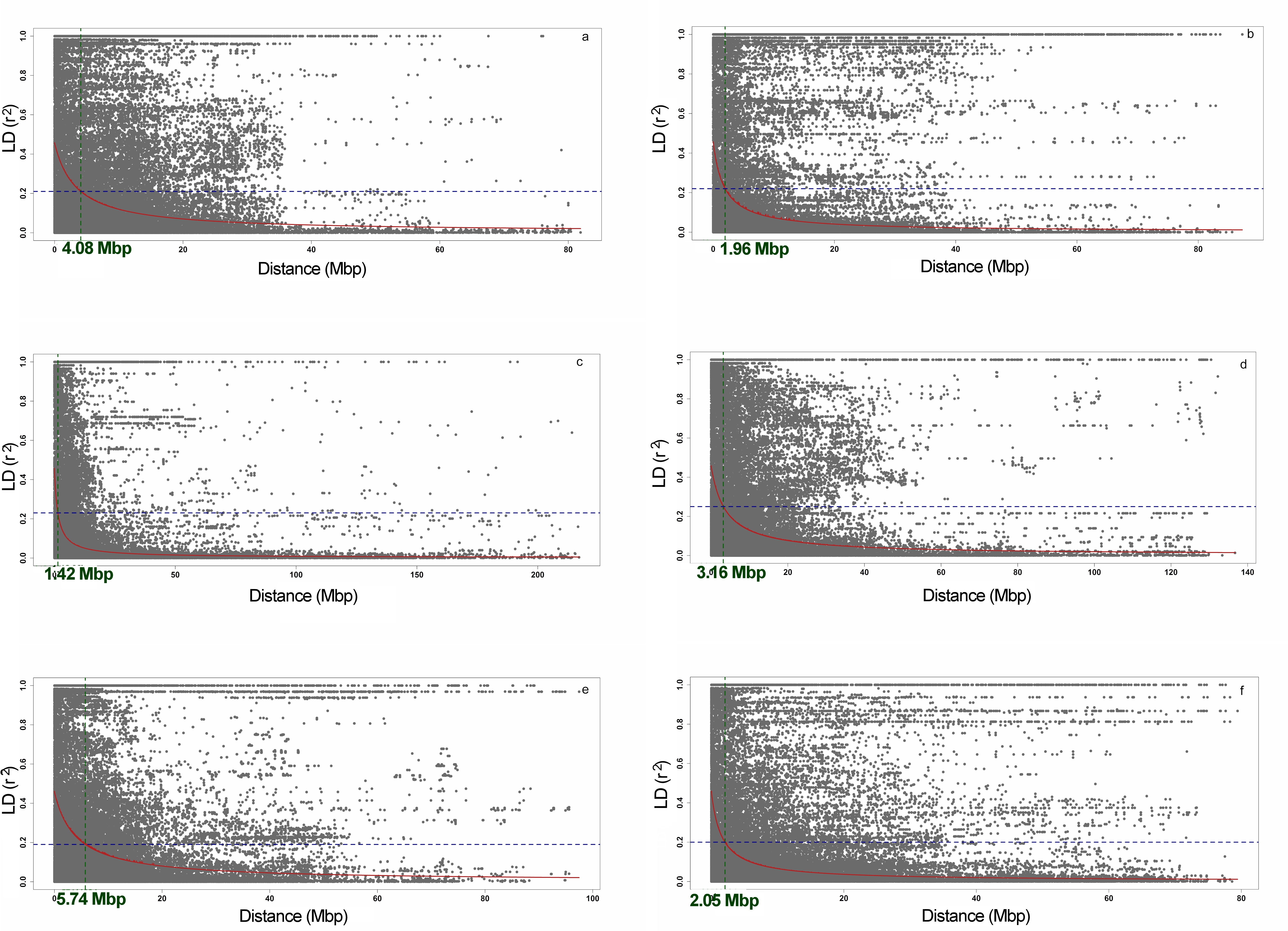

Supplement: Supplementary file 2 — Additional file 2: Supplementary Figure S1. Structure analysis in the spring wheat association-mapping panel. a) The STRUCTURE analysis showed four hypothetical subpopulations represented by different colors. b) First two components (PC1 and PC2) of a principal component analysis of the spring wheat accessions color coded by breeding program (adapted from Zhang, et al. [45]). c) Neighbor-joining phylogenetic tree showing the subpopulations corresponding to the structure analysis. Supplementary Figure S2. Quantile-quantile (QQ) plots of the observed and the expected p values of the GWAS model. a) QQ-plot for seedling resistance (infection type -IT- for the races Yr19-71 and Yr20-161); b) QQ-plot for Adult plant resistance (Disease Severity and area under disease progress curve –AUDPC-). Supplementary Figure S3. Linkage disequilibrium (LD) decay over physical distance. The scatter plots showing pairwise SNP markers LD r2 value as a function of inter-marker physical distances (Mbp) of (a) 1B chromosome; (b) 2A chromosome; (c) 3A chromosome; (d) 3B chromosome; (e) 5B chromosome; (f) 7A chromosome. The red curve represents the model fit to LD decay. The blue dashed line represents the specific critical r2 value beyond which LD is likely due to linkage. The green dashed line represents the confidence interval for the quantitative trait loci regions in which LD r2 = critical r2 value. [file 12870_2022_3916_MOESM2_ESM.zip › Additional file 2 Supplementary Figure S3 .png]
